# Mapping the Kinetic Barriers of a Large RNA Molecule's Folding Landscape

**DOI:** 10.1371/journal.pone.0085041

**Published:** 2014-02-25

**Authors:** Jörg C. Schlatterer, Joshua S. Martin, Alain Laederach, Michael Brenowitz

**Affiliations:** 1 Department of Biochemistry, Albert Einstein College of Medicine, Bronx, New York, United States of America; 2 National Evolutionary Synthesis Center, Durham, North Carolina, United States of America; 3 Department of Biology, University of North Carolina at Chapel Hill, Chapel Hill, North Carolina, United States of America; University of Leeds, United Kingdom

## Abstract

The folding of linear polymers into discrete three-dimensional structures is often required for biological function. The formation of long-lived intermediates is a hallmark of the folding of large RNA molecules due to the ruggedness of their energy landscapes. The precise thermodynamic nature of the barriers (whether enthalpic or entropic) that leads to intermediate formation is still poorly characterized in large structured RNA molecules. A classic approach to analyzing kinetic barriers are temperature dependent studies analyzed with Eyring's transition state theory. We applied Eyring's theory to time-resolved hydroxyl radical (•OH) footprinting kinetics progress curves collected at eight temperature from 21.5°C to 51°C to characterize the thermodynamic nature of folding intermediate formation for the Mg^2+^-mediated folding of the *Tetrahymena thermophila* group I ribozyme. A common kinetic model configuration describes this RNA folding reaction over the entire temperature range studied consisting of primary (fast) transitions to misfolded intermediates followed by much slower secondary transitions, consistent with previous studies. Eyring analysis reveals that the primary transitions are moderate in magnitude and primarily enthalpic in nature. In contrast, the secondary transitions are daunting in magnitude and entropic in nature. The entropic character of the secondary transitions is consistent with structural rearrangement of the intermediate species to the final folded form. This segregation of kinetic control reveals distinctly different molecular mechanisms during the two stages of RNA folding and documents the importance of entropic barriers to defining rugged RNA folding landscapes.

## Introduction

Many biological functions rely on the ability of RNA to fold into a unique three-dimensional structure. The cation-mediated folding of the *Tetrahymena thermophila* L-21 Sca I RNA ribozyme has been extensively studied as befits the first catalytic RNA to be discovered.[1], [2] Mg^2+^-mediated folding of the *Tetrahymena* ribozyme is highly sensitive to thermodynamic variables and proceeds via several parallel pathways through both short and long-lived intermediates.[3] The ribozyme's folding landscape is typically regarded as ‘rugged’ due to high barriers at some of the reaction steps.[4], [5], [6], [7] Previous studies conducted at a single temperature defined a kinetic model configuration and mapped the flux through the dominant folding pathways.[6], [8] Herein, we explore the enthalpic and entropic properties of folding of barriers to the individual steps along two of the dominate folding pathways to better understand the molecular interactions that define the transitions between reaction steps.

The temperature dependence of a reaction rate can be partitioned into enthalpic and entropic components by Eyring's transition state theory,
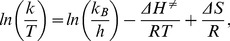
(1)where *k* is the reaction rate, *k_B_* is the Boltzmann constant, *h* is the Planck constant, Δ*S* ^‡^ is the activation entropy, Δ*H* ^‡^ is the activation enthalpy, *T* is the temperature and *R* is the gas constant.[9], [10], [11] The activation enthalpy is correlated to the energy required to break non-covalent bonds to achieve the transition state.[12] The activation entropy reflects the change in ordering of the transition state relative to the substrate. Herein we follow the change in backbone solvent accessibility at 23 distinct positions during Mg^2+^-mediated folding of the *Tetrahymena* ribozyme as a function of temperature to reveal distinct thermodynamic signatures for formation of the intermediates from the unfolded ensemble and their conversion of the to the final state.

## Results

**Figure 1:**
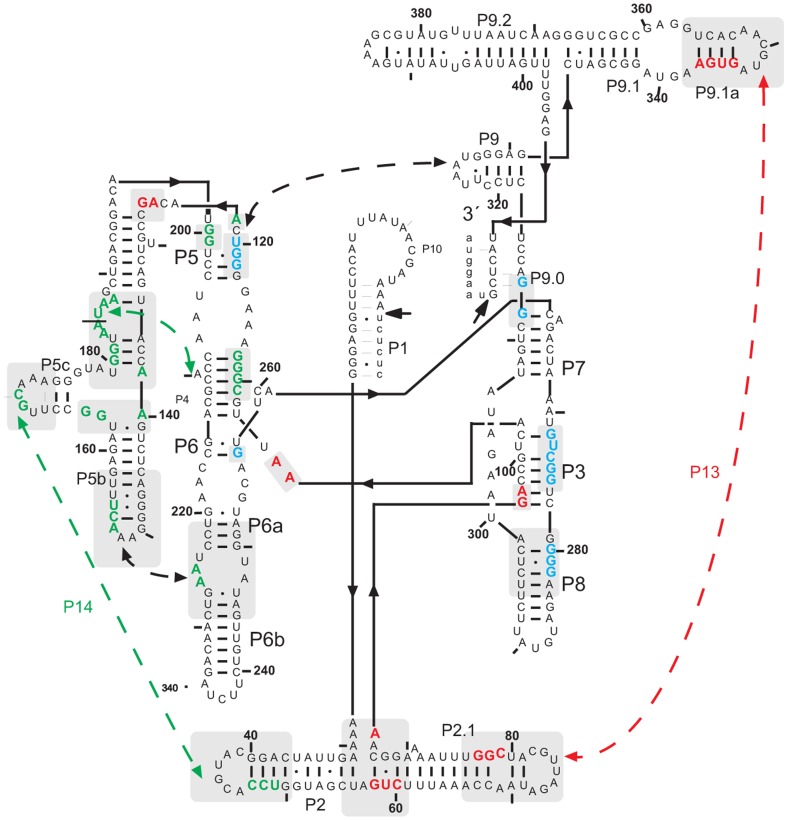
A schematic representation summarizing the secondary and tertiary structure organization of the *Tetrahymena* Sca-L21 RNA. Nucleotides colored green, red, and blue are protected from oxidation and hence solvent inaccessible in the Mg^2+^-folded ribozyme. The colors correspond the time-progress curve cluster affiliations summarized in Figure 2B. The long-range peripheral contacts are indicated with dashed arrows.

Mg^2+^-mediated folding analysis of the *Tetrahymena* ribozyme (Figure 1) was conducted at a series of temperatures from 21.5 to 51°C. The upper limit of 51°C was chosen to avoid significant native secondary structure melting. The burial of solvent accessible surfaces during folding results in diminished reactivity to a footprinting probe. This diminished reactivity is referred to as ‘protection’ and may refer to a single nucleotide or group of contiguous nucleotides whose time-dependent change in reactivity are comparable.[7], [13] The total of 23 protections were developed into time progress curves in this analysis of the temperature dependence of the Mg^2+^-mediated folding of the *Tetrahymena* ribozyme. The •OH reactivity changes of these protections were measured from 10 ms to 2 hr to define time-progress curves distributed among the different domains of the ribozyme (see *Materials and Methods*).

### Ribozyme structure and catalysis

Equilibrium •OH protection profiles are acquired in the absence and presence of Mg^2+^ in order to scale the time progress curves to fractional saturation (see *Materials and Methods*). These protection patterns were compared to determine whether temperature alters the initial or final folding states. No changes as a function of temperature were detected in the •OH reactivity profile for either the initial and final states of our folding reaction (data not shown). While these data indicate that the ribozyme's structure is invariant with temperature, our determination that the catalytically active fraction of ribozymes increases with temperature is consistent with the previously observed partitioning between an inactive conformation ‘M’ and the native ribozyme ‘N’ (Figure S2).[14], [15] As previously described elsewhere,[14] the absence of clear differences in the •OH reactivity of ‘M’ and ‘N’ likely reflect that they are topological isomers. Therefore, our data follows folding to a final state ‘F’ that is a mixture of ‘M’ and ‘N’ isomers.

### Ribozyme domains display unique temperature dependencies of folding

The locations of each of the 23 protections analyzed in this study are summarized in Figure 1. The time progress curves determined for these protections as a function of temperature are provided in the (Figure S3). The individual time progress curves typically demonstrate Arrhenius like behavior: the higher the temperature the faster the folding rate.

**Figure 2:**
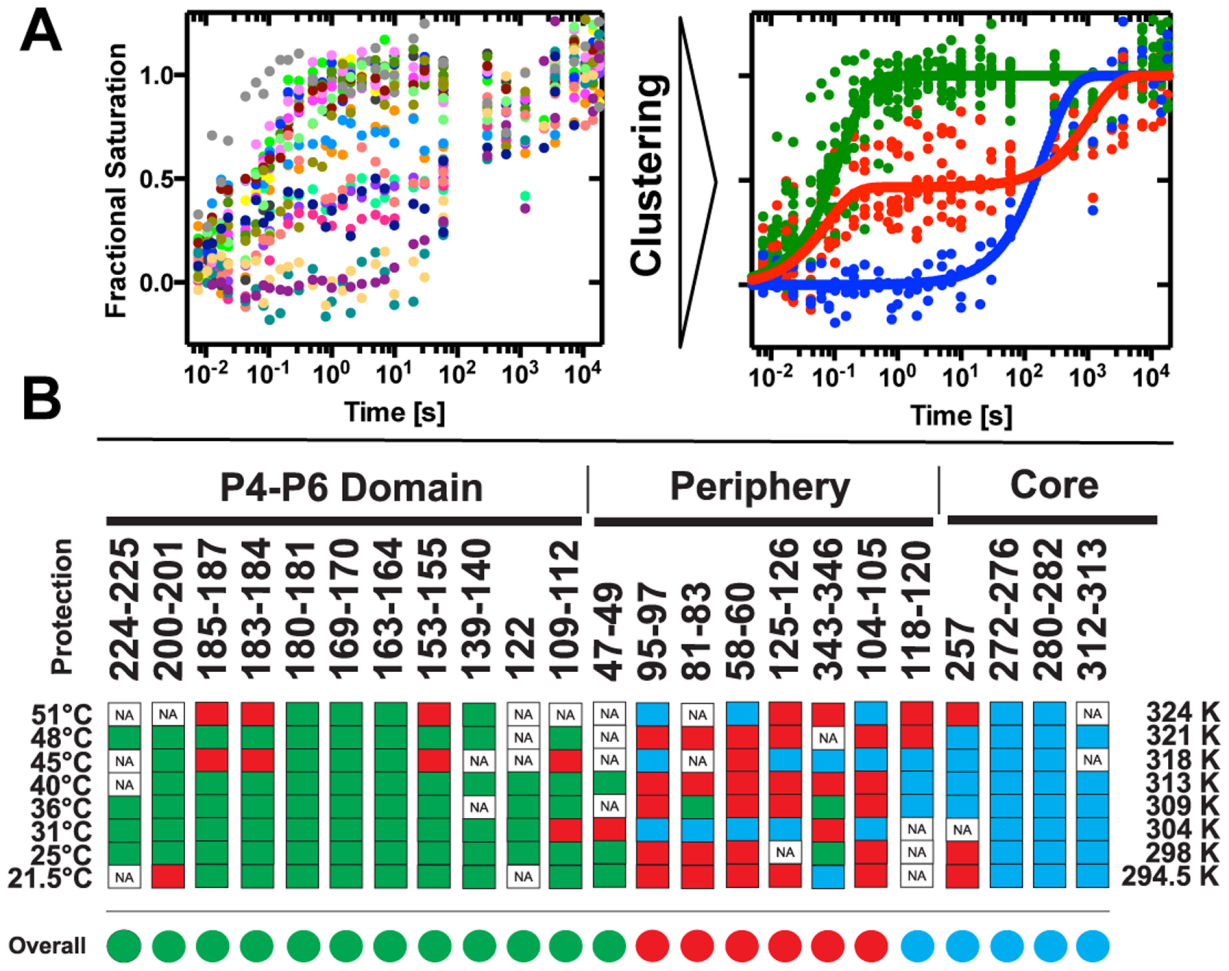
The Mg^2+^-mediated folding of the *Tetrahymena* Sca-L21 ribozyme analyzed as a function of temperature. (A) Clustering of time progression curves acquired at 25°C. Time progression curves with individual color coding (left) are associated with three statistically significant clusters (right): fast (green), intermediate (red), slow (blue). The cluster centroids are solid lines of the corresponding color. (B) Summary of temperature dependent cluster affiliation of the 23 •OH protections analyzed. Red, green, and blue rectangles correspond to the association with the fast, intermediate and slow folding cluster, respectively. The time progress curves are clustered individually at each temperature. White squares reflect data not included due to insufficient electrophoretic fragment separation (NA). The average cluster affiliations of the protections are shown as circles of the corresponding color.

In order to simplify kinetic modeling and identify whether particular parts of the ribozyme fold with unique rates, we cluster the collection of time progress curves obtained at a given temperature.[16] Three clusters are resolved at each temperature (Figure 2A; Figure S3). As seen previously,[8] the fast folding cluster (green) predominantly maps to the P4-P6 domain, the intermediate cluster (red) maps to the peripheral helices and the slow cluster (blue) maps to the catalytic core. The cluster hierarchy is temperature independent: the P4–P6 domain always folds fastest, the catalytic core slowest and the contacts of the peripheral elements are intermediate to the other two domains. However, the cluster separation decreases with increasing temperature reflecting an increase in the folding rates resulting in a decrease in the time span over which the folding reaction occurs (Figures S3 & S4).

At first glance, not all of the protections display the domain dependence generalized above. Resolution of the apparent discrepancy lies in contacts reporting inter- rather than intra-domain tertiary contacts. For example, protection 125–126 within P4–P6 maps with the medium rather than fast cluster and appears to report local structuring due to contact with the peripheral helix P9.[17], [18] Thus, it is classified as a peripheral element in Figure 2B. Nucleotide 104 contacts nucleotide 217 and nucleotide 105 forms a base triple with nucleotides 216 and 257 thereby connecting P4 with P3.[18] Protection 118–120 is within the P4–P6 domain but reports contact with the peripheral helix P9 (Figure 2B). Surprisingly, protection 118–120 is affiliated with the slow cluster, the only such behavior observed outside the catalytic core.

**Figure 3:**
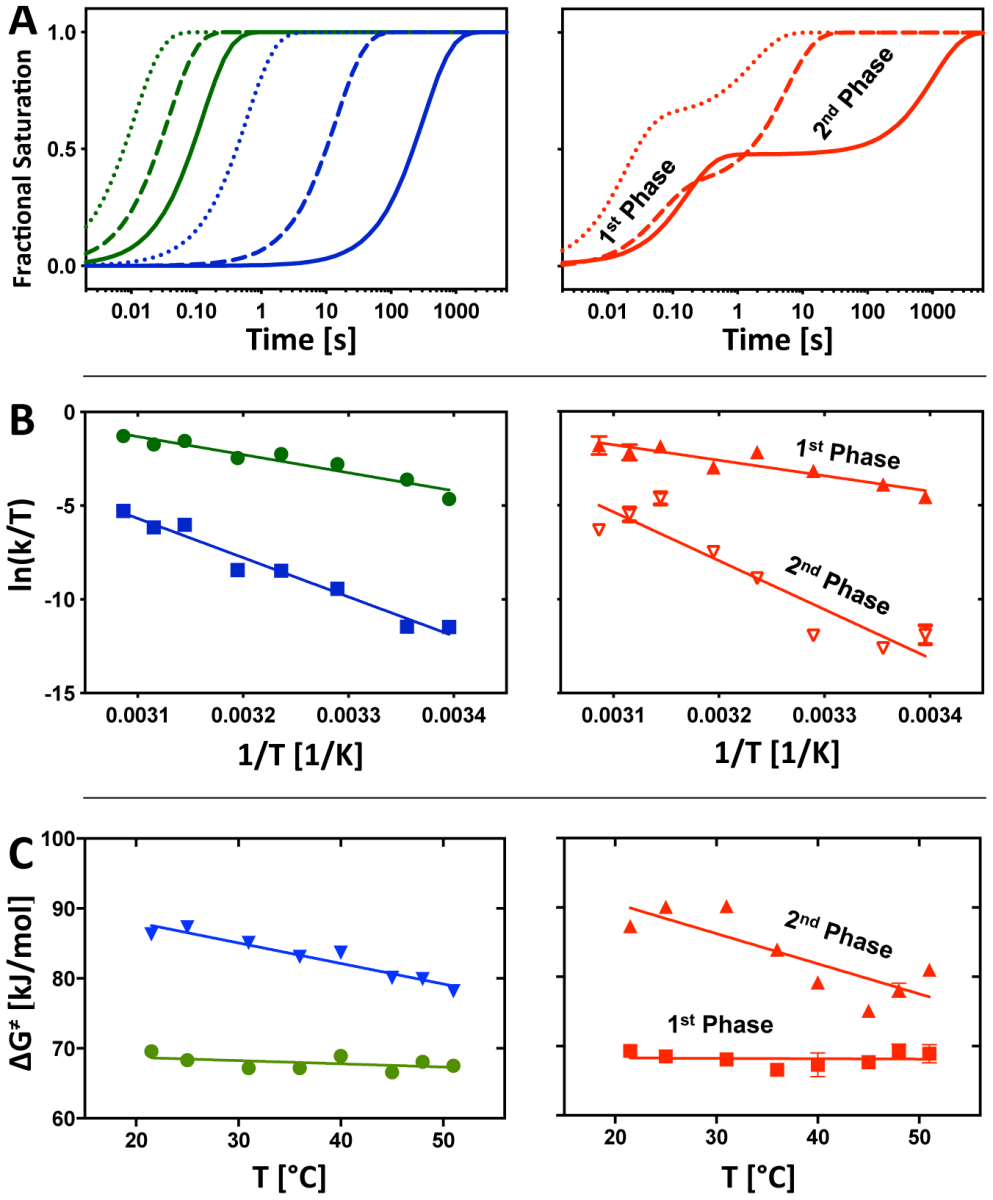
Analysis of cluster centroids. (A) The left and right panel show the simulated time progression curves derived from the global fit of cluster centroids to equation (3). The time progression of the fast (green), slow (blue), intermediate (red) cluster are shown at 25°C (solid line), 40°C (dashed line), and 51°C (dotted line). The fast and slow folding clusters are monophasic (left) whereas the intermediate cluster is biphasic. (B) The kinetic rate constants of the fast (left panel, green), slow (left panel, blue), and intermediate (right panel) cluster centroids are presented in the Eyring plots. The errors are small and visible as bars if they do not overlap the symbol. The linear fits reveal information about the entropy and enthalpy of activation during structuring of the P4–P6 domain, the core, and the peripheral elements. The free energy of activation was derived from eq. (4). Values for Δ*H* ^‡^, Δ*S* ^‡^ and Δ*G* ^‡^ are summarized in Table 1. The first and second phase of the intermediate cluster centroids are processed separately as shown in the right panel. (C) Temperature dependence of Δ*G* ^‡^. The left panel shows data points and linear fits for the fast (green circles) and slow (blue inverted triangles) cluster centroids, the right panel shows the corresponding analysis for the individual phases of the centroid associated with the medium cluster (fast phase, red squares; slow phase, red triangles).

Eyring analysis was applied to the resolved clusters in order to extract the *ΔH^‡^* and *ΔS^‡^* partitioning of the transitions. The rate constant(s) that describe each cluster was determined by fitting the cluster centroids[19] to either a single- or bi-exponential decay. A single exponential decay describes the fast (green) and slow (blue) while a bi-exponential decay describes the medium (red) cluster at all of the temperatures analyzed (Figure S5). Figure 3A illustrates the different temperature behaviors of the clusters by showing the curves fit to the cluster centroids at 25°C (solid line), 40°C (dashed line), and 51°C (dotted line.) While folding is faster as the temperature elevates for all three clusters, the slow cluster accelerates more compared to the fast cluster. The relative amplitudes of the two phases of the medium cluster do not track with temperature (Figure S6).

The differences described qualitatively above are reflected in the values resolved from the Eyring analysis of the clusters (eq. 1; Figure 3B). The two phases of the medium cluster was separately analyzed. The activation energies (Δ*G* ^‡^) resolved for the slow cluster is greater than that for the fast cluster showing that the energetic barrier to folding the catalytic core is greater than that for P4–P6 (Table I). Δ*G* ^‡^ resolved for the fast and slow phases of the intermediate cluster closely match the Δ*G* ^‡^ values resolved for P4–P6 and the catalytic core, respectively. As is explored more fully in the *Discussion*, this observation is consistent with the view that P4–P6 serves as a scaffold for the initial organization of the peripheral contacts but that final structuring of the periphery occurs in concert with folding of the catalytic core. The partitioning of Δ*G* ^‡^ between Δ*H* ^‡^ and Δ*S* ^‡^ differs dramatically between the fast and slow clusters (Table 1). In comparison, Δ*H* ^‡^ for the slow cluster doubles whereas Δ*S* ^‡^ changes more than seven fold.

**Table 1:** The values of Δ*G* ^‡^, Δ*H* ^‡^ and Δ*S* ^‡^ resolved from the three clusters of time-progress curves shown in Figures 2A and S4.

*Cluster Centroid*	Δ*H* ^‡^ [kJ/mol]	Δ*S* ^‡^ [kJ/molK]	Δ*G* ^‡^ [kJ/mol]
**P4-P6 (green)**	80±10	0.040±0.031	69.6±0.1 (21.5°C) 67.5±0.2 (51°C)
**Periphery, Phase 1 (red)**	63±12	−0.019±0.038	69.3±0.3 (21.5°C) 68.9±1.3 (51°C)
**Periphery, Phase 2 (red)**	189±29	0.336±0.094	87.3±0.1 (21.5°C) 81.0±0.6 (51°C)
**Catalytic Core (blue)**	173±13	0.294±0.042	86.3±0.2 (21.5°C) 78.2±0.1 (51°C)

The two kinetic phases of the ‘red cluster’ were independently analyzed. The Δ*G* ^‡^ values determined for the lowest and highest temperatures investigated are shown.

### A common kinetic model describes folding at every temperature

The analysis of the progress curve clusters described above provides insight into the hierarchical folding of the *Tetrahymena* ribozyme and the thermodynamic nature of the reaction barriers to each step in the hierarchy. In previous papers we determined the kinetic model configuration for folding of the *Tetrahymena* ribozyme at 25°C that provides quantitative insight into the dominant folding pathways and the number and nature of the folding intermediates.[6] The Kinfold software exhaustively tests all the kinetic model configurations consistent with the number of progress curve clusters. Kinfold then determines the optimum mapping of intermediates and rates to time progress curve clusters.[19] The reverse rates are bound to zero in our fitting since the folding reaction is initiated with an excess of Mg^2+^; this reduces the number of parameters to fit and thus improves stability of the fitting procedure.

**Figure 4:**
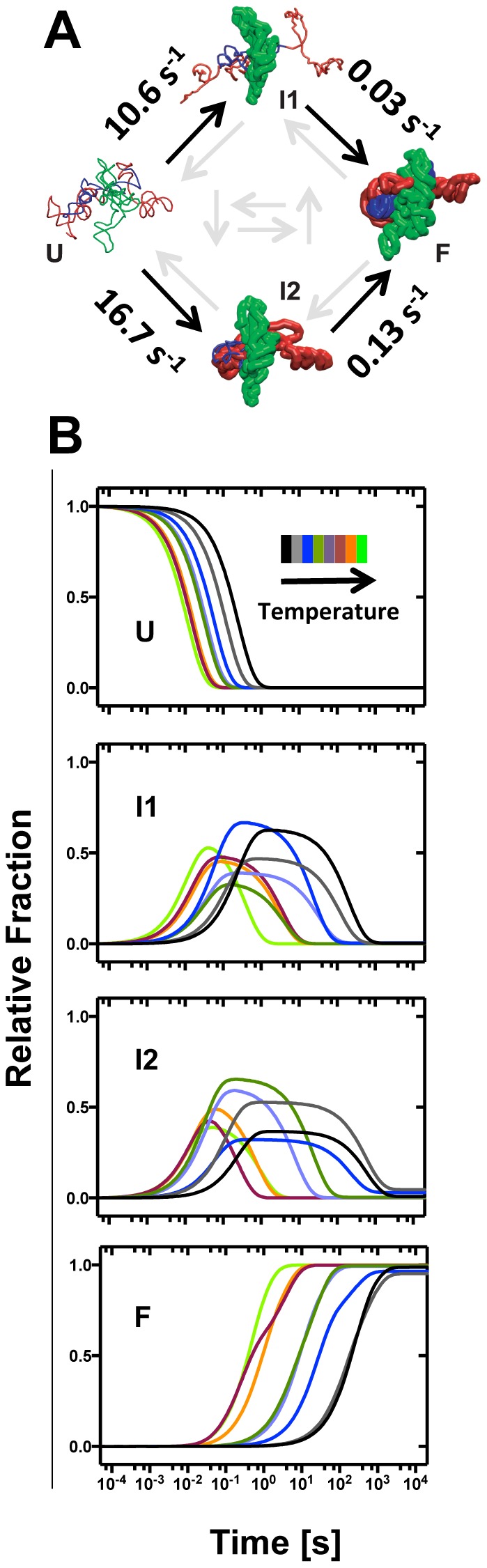
The structural-kinetic model common to all of the analyzed temperatures that was resolved for the Mg^2+^-induced folding of the *Tetrahymena* ribozyme. (A) The model configuration includes the unfolded (U), and folded (F) states, and two intermediates (I1 and I2). The structured regions of each species are modeled from the •OH reactivity patterns and rendered as bold ribbons. The kinetic rate constants are determined for all forward reactions (black arrows) by constraining all other folding reaction (grey arrows) as indicated in the *Materials and Methods*. The folding rates determined at 40°C are shown (s^−1^). (B) From the top to the bottom, the calculated time evolution of U, I1, I2 and F are shown from 21.5 and 51°C using the color bar insert to the top panel.

The kinetic model previously identified for the *Tetrahymena* ribozyme[8] also describes all of the analyzed temperatures (Figure 4A). The model consists of four species: the unfolded RNA (U), the folded molecule (F) and two intermediates, I1 and I2. P4–P6 is exclusively structured in I1. P4–P6 and the peripheral helices are structured in I2. The study examines the difference in the thermodynamic signature of the two dominant folding routes [16]; U → I1 → F and U → I2 → F. Therefore only the k-values for the reactions U → I1, U → I2, I1 → F, I2 → F were determined as described in the *Materials and Methods*. The rate constants resolved at 40°C, approximately the middle of the analyzed range, are shown in Figure 4A to give a sense of the measured folding rates. The rates resolved at the other temperatures are presented in Table S2.

### Intermediate formation is differentially temperature dependent

The kinetic model revealed partitioning of the folding flux between two intermediates from the earliest steps of folding. This behavior is temperature independent. The rates at which the intermediates are formed (U → I1 and U → I2) are two orders of magnitude faster than the rates at which there are resolved to the final form (I1 → F and I2 → F; Figure 4A). Interestingly, the relative rates resolved for U → I1 and U → I2 are temperature dependent (Figure 4A). At 40°C, the rate of U → I1 is half that of U → I2. This relationship is reversed at low temperature (Table S2).

Calculation of the time evolution of the folding species U, I1, I2 and F from the best-fit parameters clearly shows the temperature dependence of this folding reaction (Figure 4B). U disappears faster with increasing temperature; an order of magnitude separates the rates by which U folds between the lowest and highest temperatures. Similarly, the rate of F formation increase with temperature although in this case three orders of magnitude separate the curves calculated at the lowest and highest temperatures. As demanded by mass conservation, the difference in the temperature dependence of U disappearance and F formation is reflected in a decrease in both intermediates' lifetimes and populations with increasing temperature. At low temperatures both intermediates display the elongated lifetime characteristic of kinetic trapping (Figure 4B; black curves) while at high temperatures they approach the transient behavior characteristic of a freely folding on-pathway intermediate (Figure 4B; lime green curves).

### Enthalpy and entropy of activation allows partitioning of folding species

**Figure 5:**
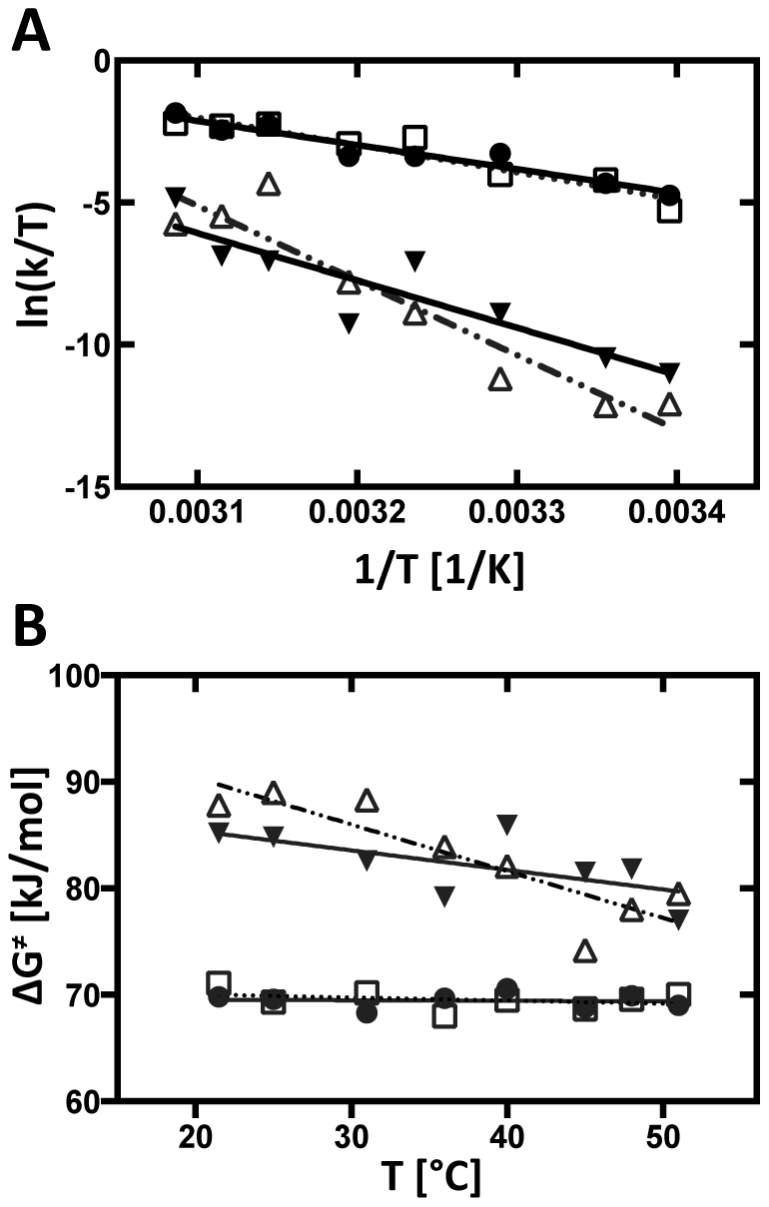
Eyring analysis of the reactions for the structural-kinetic model as depicted in Figure 4A. Values for Δ*H* ^‡^, Δ*S* ^‡^ and Δ*G* ^‡^ are summarized in Table 2. (A) Eyring plots were fit to the Eyring equation (eq. 1). Eyring plots for the primary transitions U → I1 (full circles), U → I2 (empty square) and secondary transitions I1 → F (solid triangles), I2 → F (empty triangles) are shown. (B) Temperature dependence of Δ*G* ^‡^. Δ*G* ^‡^ values are derived by solving eq. 4 and fitted linearly; U → I1 (full circles), U → I2 (open squares), I1 → F (solid triangles) and I2 → F (open triangles).

Figure 5A shows Eyring plots of the rate constants; the slope and the y-intercept of linear fits of the individual reactions of the kinetic models yield, respectively, the enthalpy and entropy of activation (Eq. 1; Table 2). The transition activation enthalpies affiliated with the primary reactions U → I1 and U → I2 are roughly half of those affiliated with the secondary reactions I1 → F and I2 → F (Table 2). The entropies of activation of the secondary transitions are an order of magnitude larger than those affiliated with U → I1 and U → I2. The free energy of activation for the primary transitions is consistently smaller than those of the secondary transitions. Figure 5B shows the significant temperature dependency of Δ*G* ^‡^ for the secondary transitions.

**Table 2:** The values of Δ*G* ^‡^, Δ*H* ^‡^ and Δ*S* ^‡^ resolved for the individual steps of the two dominant pathways of structural kinetic model, U → I1 → F and U → I2 → F.

*Transition*	Δ*H* ^‡^ [kJ/mol]	Δ*S* ^‡^ [kJ/molK]	Δ*G* ^‡^ [kJ/mol]
**U → I1**	70±8	0.003±0.026	69.8±0.1 (21.5°C)
			69.0±0.1 (51°C)
**U → I2**	80±10	0.032±0.035	71.1±0.1 (21.5°C)
			70.0±0.1 (51°C)
**I1 → F**	138±27	0.182±0.089	85.2±0.1 (21.5°C)
			77.0±0.1 (51°C)
**I2 → F**	218±30	0.438±0.096	87.8±0.1 (21.5°C)
			79.5±0.1 (51°C)

The Δ*G* ^‡^ values determined for the lowest and highest temperatures investigated are shown.

## Discussion

Our study explores the activation energies of discrete steps during the Mg^2+^-mediated folding of the *Tetrahymena* ribozyme from the formation of the earliest detectable specific contacts. The activation barriers that we describe differ not only in the heights but also in their thermodynamic nature. We first compared the time-progress curve clusters that describe tertiary structure formation of the P4–P6 domain, the peripheral helices and the catalytic core. We observe much greater barriers to the latter folding steps compared to the initial folding reactions in agreement with single molecule characterizations of the folding pathways of the group I introns.[20], [21] We also determined the activation energies for the reactions within the dominant folding pathways resolved by kinetic modeling. The later analysis reveals distinct barriers for the resolution, but not formation of the folding of the two intermediates species.

Three statistically significant time-progress curve clusters were resolved at all of the temperatures investigated. The P4–P6 domain (green) always folds first, predominantly followed by the peripheral elements (red), and finally the catalytic core (blue; Figure 2 & Figure S3). Thus, temperature does not alter the mechanism by which the *Tetrahymena* ribozyme folds but rather modulates the reaction barriers. Higher temperatures monotonically lead to faster folding rates (Figure 3A) resulting in linear Eyring plots (Figure 3B). The values of Δ*H* ^‡^ and Δ*S* ^‡^ resolved for the P4–P6 domain (80±10 kJ/mol and 0.040±0.031 kJ/mol.K, respectively; Table 1) differ from those resolved for the isolated pyrene-labeled P4–P6 domain under similar, but not identical conditions (Δ*H* ^‡^ ≈110 kJ/mol and Δ*S* ^‡^ ≈0.13 kJ/mol.K).[22] While these differences could result from the nonidentity of the folding conditions or the different folding assays, the observation of a higher enthalpic barrier suggests that contacts with the peripheral helices facilitate P4–P6 folding. The slightly higher entropic penalty to folding P4–P6 in the context of full length RNA could be due to concurrent formation of tertiary contacts (Figure 2A). However, these results cannot exclude that truncation not only removes energetically favorable contacts in the full length RNA but may also lead to an expansion of the denatured-state ensemble by weakening transient residual structure.

The barrier to folding the catalytic core is much greater than that for the P4–P6 scaffold, making this transition the rate limiting step in the folding of the intron (Δ*H* ^‡^ = 173±13 kJ/mol and Δ*S* ^‡^ = 0.294±0.042 kJ/mol.K; Table 1). In contrast to the P4–P6 domain, in which Δ*G* ^‡^ changes only slightly with temperature, ΔG^‡^ for catalytic core formation decreases dramatically by 8.1 kJ/mol over the temperature range studied (Figure 3C, left panel). Our value of Δ*G* ^‡^ = 83.1±0.2 kJ/mol at 36°C is in good agreement with the results of a oligonucleotide hybridization assays Δ*G* ^‡^ = 85.81±3.34 kJ/mol at 37°C albeit at a lower salt folding conditions.[23] That the enthalpic and entropic components are higher in our analysis, Δ*H* ^‡^ is roughly doubled and Δ*S* ^‡^ is tenfold higher (ΔH^‡^ ≈96 kJ/mol and ΔS^‡^ ≈0.034 kJ/mol.K)[23], suggests that salt likely affects the entropy – enthalpy compensation underlying the barrier to catalytic core structuring from the folding intermediates.

Perhaps the most intriguing result is the biphasic nature of the cluster assembled from contacts of the peripheral helices that clearly reflect its ‘intermediary role’ between the folding of the P4–P6 ‘scaffold’ and the catalytic core. The tertiary contacts included in this cluster report the folding progress of the P14 and P13 helices and the P5–P9 contact. The biphasic progression is present throughout the temperature range analyzed and this suggests that there may be two folding populations. In this view, one population folds in concert with P4–P6 while the second population folds with the catalytic core. This is in agreement with the two intermediate, parallel folding model proposed for the *T. thermophila* group I intron.[6], [8] However, further study is required to fully distinguish this hypothesis from sequential progression in which the kinetic phases reflects partial saturation of each tertiary contact.

The free energy of activation of the first periphery phase is comparable to that of P4–P6 and likewise changes only little with temperature (Table 1; Figure 3C). The constituent values of Δ*H* ^‡^ and Δ*S* ^‡^ are both lower than those of P4–P6 indicating that the energetics of formation of these interactions is not identical to that of P4–P6. The energetics of the second periphery phase is identical (within experimental error) to that of the catalytic core (Table 1; Figure 3C). The relative amplitudes of the two peripheral phases does not vary systematically with temperature; an observation for which we do not have an explanation.

To further explore the energetic partitioning of the activation energy of each step in the folding reaction we resolved the kinetic model at each temperature analyzed; the two-intermediate model with parallel pathways[8] describes each folding reaction (Figure 4A). Thus, temperature partitions the flux among the observed folding pathways without altering the underlying folding pathways. While thermodynamic parameters could not be resolved for all of the reactions, we determined them for the steps of the two dominant pathways, U → I1 → F and U → I2 → F.[16]

The values of Δ*H* ^‡^ and Δ*S* ^‡^ resolved for the primary transitions, U → I1 and U → I2, are identical within experimental error (Table 2). This result is at first glance surprising. The folding of only P4–P6 contacts constitute I1 while I2 is composed of both the P4–P6 contacts and those of the periphery. Clearly, P4–P6 folding is rate limiting to the formation of I2. This result is consistent with the view that P4–P6 serves as a folding scaffold.

In contrast, the values of Δ*H* ^‡^ and Δ*S* ^‡^ resolved for the secondary transition I1 → F are dramatically less than those of the parallel secondary transitions I2 → F. This result is consistent with the much longer lifetime of I2 compared to I1 and implies that premature structuring of peripheral contacts is not favorable to fast folding and thus, that I2 is misfolded. That Δ*S* ^‡^ is greater for the latter reaction is also consistent with studies showing that an intermediate structure is topologically misfolded.[14] A plausible interpretation of this observation is that the barrier to simultaneously folding the periphery and catalytic core (*i.e.*, the I1 → F transition) is lower than *refolding* the periphery and folding the catalytic core (*i.e.*, the I2 → F transition). The more favorable entropy change partially offsets the larger enthalpic barrier.

While RNA folding is an energetically complex process, the formation and breaking of hydrogen bonds typically plays an important role. Enthalpy/entropy compensation implies major contributions from biopolymer-solvent interactions.[24] Formation of an isolated hydrogen bond requires ∼23 kJ/mol [25] and the relative probability of a potential hydrogen bond being completely unsatisfied (either by an intramolecular partner or by water) is very small, 2×10^−4^ at room temperature.[26] Thus, even a single unsatisfied hydrogen bond is unlikely to persist for long during folding.

Formation of a tertiary hydrogen bond in RNA molecules is reported to require 2–4 kJ/mol.[27], [28], [29], [30], [31] In addition, the hydrogen bond donors and acceptors might already participate in non-covalent interactions in a local but not tertiary setting. Thus, the formation of a tertiary hydrogen bond during RNA folding roughly is affiliated with ≤4 kJ/mol. This value is less then our measured enthalpies of activation, suggesting that folding to the F state requires the breaking and subsequent formation of multiple hydrogen bonds.

The initial state, U, in these studies is a relatively compact ensemble [32] of unfolded conformations that possess the majority of the native secondary structure elements. All other hydrogen bond donors and acceptors of the RNA presumably participate in intramolecular hydrogen bonds or with water. Using 4 kJ/mol as an upper bound for the energetic cost of breaking a hydrogen bond in the transition state, the primary reactions, U → I1 and U → I2 involve 18–20 hydrogen bonds, much less than for the secondary reactions I1 → F (35 hydrogen bonds) and I2 → F (54 hydrogen bonds). In this view, I2 clearly requires greater restructuring compared with I1 to reach the final folded form. While this calculation oversimplifies the folding energetics, it does provide a way to visualize the relative complexity of the individual folding steps in terms of bonds broken and formed.

In summary, Eyring analysis of the Mg^2+^-mediated folding of the *Tetrahymena* ribozyme partitions the energetic barriers for early and late steps of the folding reaction into their entropic and enthalpic contributions. While temperature uniformly increases the folding rate within a constant folding mechanism, it does redistribute the folding flux among the dominant reaction pathways. The barrier to forming P4–P6 is exclusively enthalpic within experimental error. In contrast, the substantial barrier to catalytic core folding is offset by favorable entropy. The organization of the peripheral helices follows a biphasic progression; the phases of which track with P4–P6 and the catalytic core, respectively. The barriers resolved for the primary transitions (U → I1 and U → I2) by kinetic modeling are energetically equivalent despite the fact that I2 includes structuring of the peripheral helices. The differences in the barriers resolved for the secondary transitions are rationalized by the I2 intermediate being topologically misfolded.

Our Eyring analysis of the time-progress curves and the kinetic modeling of a folding RNA yield consistent conclusions (and values) for the energetic barriers that provide insight into the energetics of both the early and late folding steps and the consequences of the partitioning of the folding reaction at its onset into parallel pathways. The consistency between these approaches suggests that bulk temperature studies have a robustness that could, and should be, applied to other folding reactions.

## Materials and Methods

### RNA preparation

*Tetrahymena* ribozyme RNA was prepared by *in vitro* transcription of *Sca* I-cut pT7L-21 DNA and purified as previously described.[33] The RNA was labeled at either the 5′ end with [γ-^32^P]ATP using bacteriophage T4 polynucleotide kinase or the 3′ end with [α-^32^P]dCTP using Klenow fragment. The labeled RNA was purified by electrophoresis through 7 M urea/4% polyacrylamide gels, extraction, precipitation and resuspension as described.[34]

### Time-resolved •OH footprinting

The Mg^2+^-mediated folding of the *Tetrahymena* ribozyme was followed in buffer containing 100 mM KCl, 10 mM sodium cacodylate and 0.1 mM EDTA (pH 7.4) at the temperatures of interest: 21.5, 25, 31, 36, 40, 45, 48, and 51°C. Folding was initiated by the addition of 10 mM MgCl_2_. Fast Fenton footprinting experiments were carried out as described using a KinTek® RQF-3 three-syringe mixer for folding times of up to 1 min.[35] Time points longer than 1 min and less than 2 hr were sampled by hand mixing using a standard peroxidative hydroxyl radical footprinting protocol.[36]

### Data Analysis

Each progress curve was scaled to fractional saturation,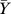, by
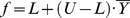
(2)where *f* denotes the integrated density of the bands comprising a protection. The lower limit to the transition, *L*, was determined from samples lacking MgCl_2_. The upper limit, *U*, was collected on fully folded samples 10 mM MgCl_2_. This scaling of the progress curves allows multiple data sets, including rapid-mix and hand-mix experiments, to be combined into a single data set.

### Time-progress curve clustering

k-means clustering with a Manhattan distance metric implemented in Matlab 7.5 (The Mathworks, Natick MA) was used to cluster the scaled progress curves.[6] The statistically significant number of clusters was determined using the Gap Statistic [37] which analyzes the relative within cluster dispersion (*W_k_*) as a function of the number of clusters and determines the *k* value where *W_k_* decreases linearly.[6] The resolved cluster centroids determined as a function of temperature were fit to equation (3) in Origin 6.1 (OriginLab):
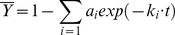
(3)where *α_i_* and *k_i_* are the amplitude and rate constant, respectively, of the *i* ^th^ kinetic phase. The reaction rate constants, *k*, and the temperature, *T*, are plotted as ln(*k*/*T*) versus 1/*T* and fit to the Eyring equation, eq. (1). The activation enthalpy is determined by the slope while the entropy of activation is determined by the intercept of the ln(*k*/T) axis. The error in the resolved parameters is propagated in the usual fashion. The Gibbs energy of activation, Δ*G* ^‡^, is directly determined from the kinetic rates and their errors according to



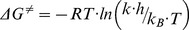
(4), where *k* is the reaction rate constant, *k_B_* is the Boltzmann constant, *h* is the Planck constant, *T* is the temperature in Kelvin and *R* is the gas constant.

### Structural - kinetic modeling

The cluster centroids were used as time-progress curves to determine the best kinetic model and values using the KinFold v2.0 software (Figure S1).[19] Briefly, the kinetic model was established by iteratively solving the coupled linear differential equation
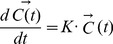
(5)where *K* is the square matrix containing the rate constants between species and ***C*** *(t)* is the vector containing the concentrations of the individual species in solution at any given time, t. The solution to Eq. 5 is accomplished by standard mathematical techniques. All possible mappings of the intermediate curves to the time-progress curves are enumerated and the best fitting model chosen based on root mean square error (*RMSE*) to the cluster centroids.[19] The errors in the kinetic model parameters were estimated using a standard bootstrap procedure. The k-values for the reactions U → I1, U → I2, I1 → F, I2 → F were determined by constraining all of the other forward and reverse rates to a minimal value of 0.00001 s^−1^.

## Supporting Information

Figure S1 **A roadmap for the experiments and analysis that constitute this study.**
(PDF)

Figure S2 **Measurement as a function of temperature of the fraction of L-21 Sca I RNA that is in its native, catalytically active conformation determined by standard activity assays^1^.** The experiments were conducted in triplicate and averaged. The native fraction was normalized to the 51°C data. Error bars for 25, 31, and 36°C data overlap with the symbol.
(PDF)

Figure S3 **Clustering of time progression curves from experiments conducted between 21.5°C and 51°C.** Time progression curves with individual color coding (left) are associated with three statistically significant clusters (right): fast (green), medium (red), slow (blue).
(PDF)

Figure S4 **Mean cluster centroids for folding of the L-21 Sca I RNA at the eight temperatures analyzed.** The fast, medium and slow folding clusters are shown in green, red and blue, respectively.
(PDF)

Figure S5 **Fitting of the medium cluster.** Red dots indicate the raw data of the cluster centroid, the black line shows the bi-exponential fit of the data to equation 3.
(PDF)

Figure S6 **Amplitudes of first (red) and second (green) phase of the intermediate cluster centroid.**
(PDF)

Table S1 **Rates of progression of the fast, slow and medium cluster centroids at different temperatures.**
(PDF)

Table S2 **Rates of conversion between folding species U, I1, I2, F at different temperatures.**
(PDF)

Table S3 **Errors for the standard fitting models according to Martin et al.** Briefly, errors are calculated by summing how far each data set goes below zero and normalizing according to how many points are below zero. Model 2 features the smallest error and reflects the most likely model configuration that includes U, F, and two folding intermediates (I1 and I2).
(PDF)
