# Supplementary material for: Mapping the Kinetic Barriers of a Large RNA Molecule's Folding Landscape
Source: PLoS One. 2014 Feb 25;9(2):e85041. doi: 10.1371/journal.pone.0085041 (PMC3934814; doi:10.1371/journal.pone.0085041)
Supplement: Figure S1 — A roadmap for the experiments and analysis that constitute this study. (PDF) [file pone.0085041.s001.pdf]

Supporting Information, **Figure S1**
Title: Mapping the kinetic barriers of a large RNA molecule's folding landscape
Authors: Jörg C. Schlatterer, Joshua S. Martin, Alain L. Laederach, Michael Brenowitz

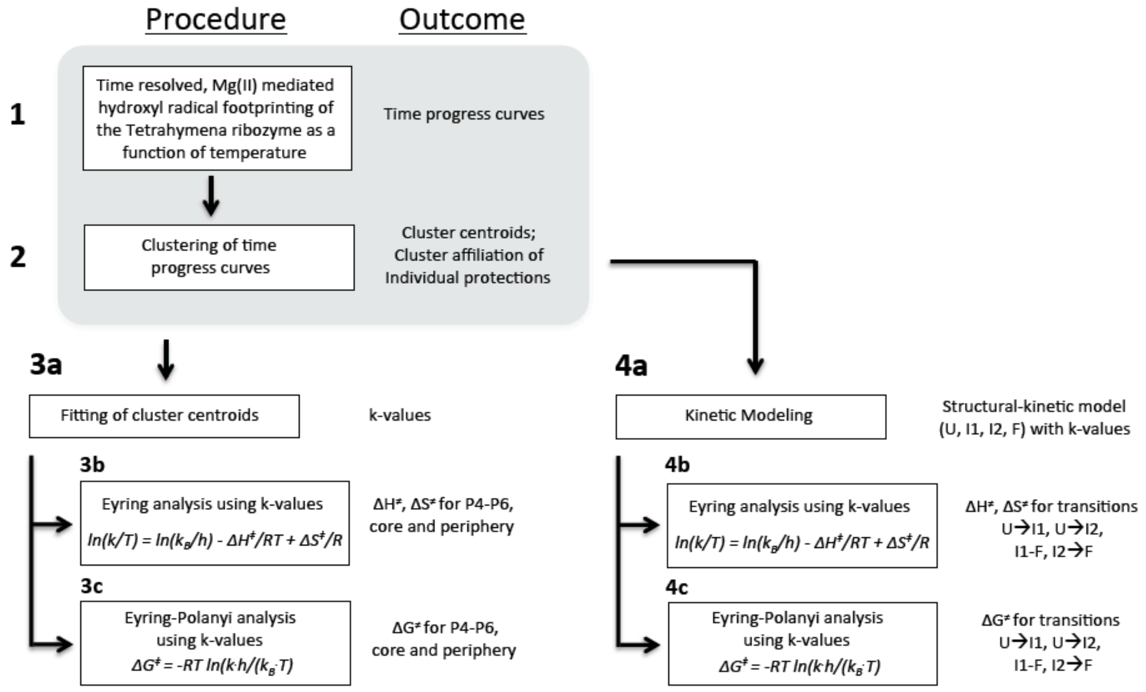

Figure S1. Roadmap for the study "Mapping the kinetic barriers of a large RNA molecule's folding landscape".
