# Supplementary material for: Mapping the Kinetic Barriers of a Large RNA Molecule's Folding Landscape
Source: PLoS One. 2014 Feb 25;9(2):e85041. doi: 10.1371/journal.pone.0085041 (PMC3934814; doi:10.1371/journal.pone.0085041)
Supplement: Figure S2 — Measurement as a function of temperature of the fraction of L-21 Sca I RNA that is in its native, catalytically active conformation determined by standard activity assays1. The experiments were conducted in triplicate and averaged. The native fraction was normalized to the 51°C data. Error bars for 25, 31, and 36°C data overlap with the symbol. (PDF) [file pone.0085041.s002.pdf]

Supporting Information, **Figure S2**
Title: Mapping the kinetic barriers of a large RNA molecule's folding landscape
Authors: Jörg C. Schlatterer, Joshua S. Martin, Alain L. Laederach, Michael Brenowitz

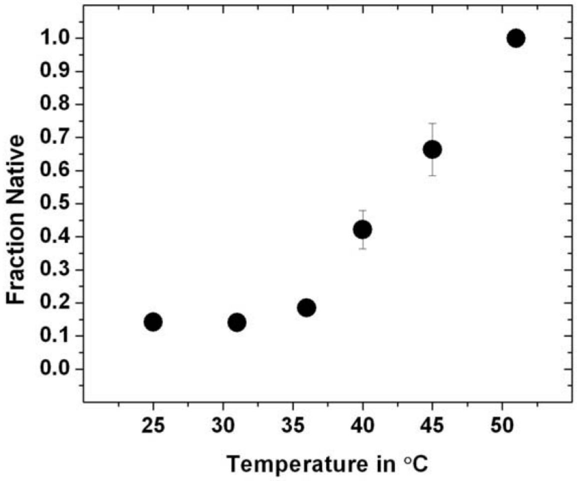

Figure S2. Fraction native of the L-21 Sca I RNA at a given temperature determined by standard activity assays[1]. All experiments were repeated three times and averaged. Fraction native was normalized to the 51°C data. Error bars for 25, 31, and 36°C data overlap with the symbol.

(1) Russell, R.; Herschlag, D. *J Mol Biol* 1999, *291*, 1155.
