# Supplementary material for: Mapping the Kinetic Barriers of a Large RNA Molecule's Folding Landscape
Source: PLoS One. 2014 Feb 25;9(2):e85041. doi: 10.1371/journal.pone.0085041 (PMC3934814; doi:10.1371/journal.pone.0085041)
Supplement: Figure S3 — Clustering of time progression curves from experiments conducted between 21.5°C and 51°C. Time progression curves with individual color coding (left) are associated with three statistically significant clusters (right): fast (green), medium (red), slow (blue). (PDF) [file pone.0085041.s003.pdf]

Supporting Information, **Figure S3**
Title: Mapping the kinetic barriers of a large RNA molecule’s folding landscape
Authors: Jörg C. Schlatterer, Joshua S. Martin, Alain L. Laederach, Michael Brenowitz

Time progression curves of local probes

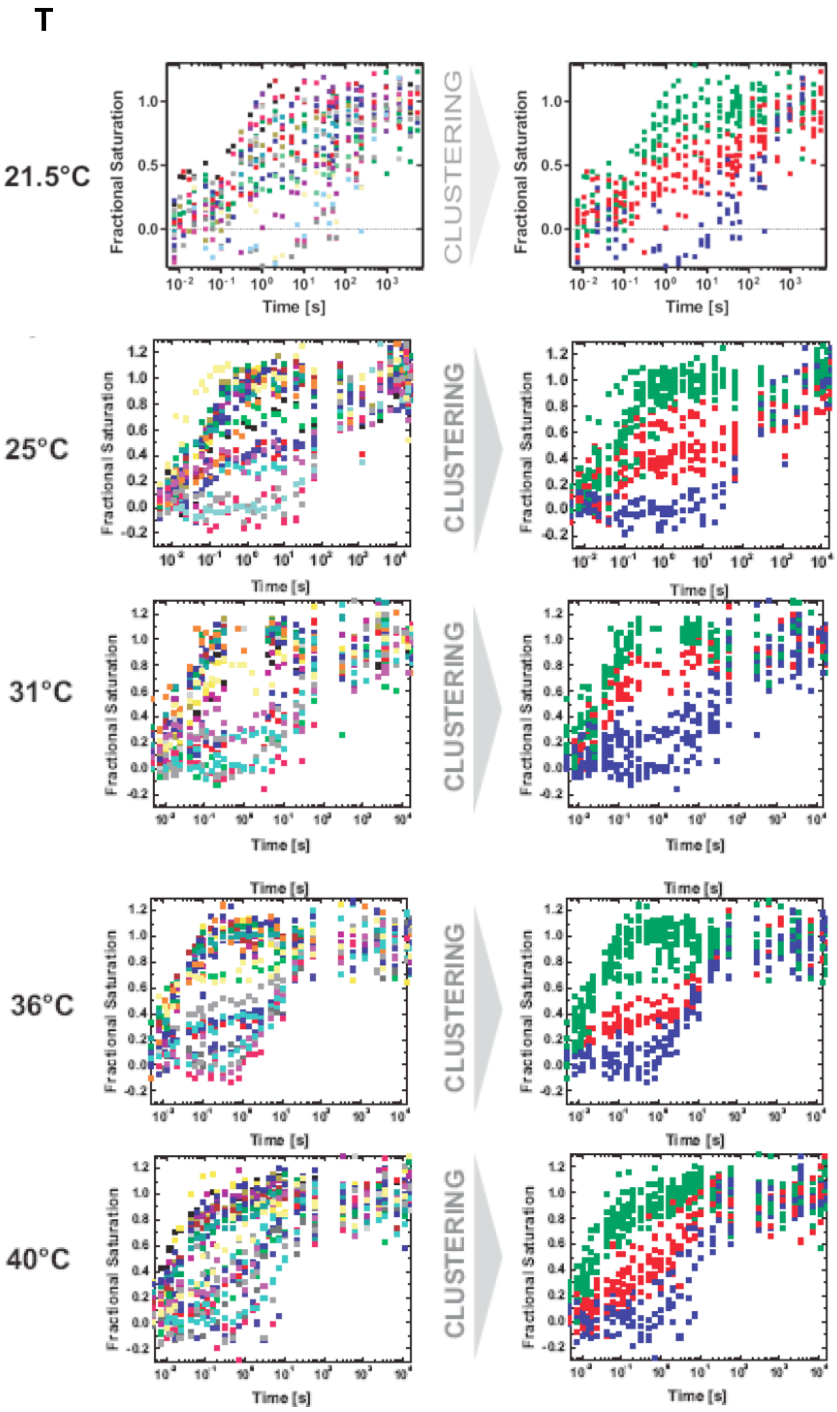

Color code for time progression curves in the left column

Protection (nucleotide)	Color
47-49	Black
58-60	Red
81-83	Green
95-97	Blue
104-105	Cyan
109-112	Magenta
118-120	Dark gray
122	Yellow
125-126	Medium gray
139-140	Dark yellow
153-155	Navy
163-164	Light gray
169-170	Purple
180-181	Wine
183-184	Olive
185-187	Dark cyan
200-201	Royal
224-225	Orange
257	Violet
272-276	Pink
280-282	Gray
312-313	Light cyan
343-346	Light yellow

Folding cluster affiliation of time progression curves in the right column

FAST
MEDIUM
SLOW

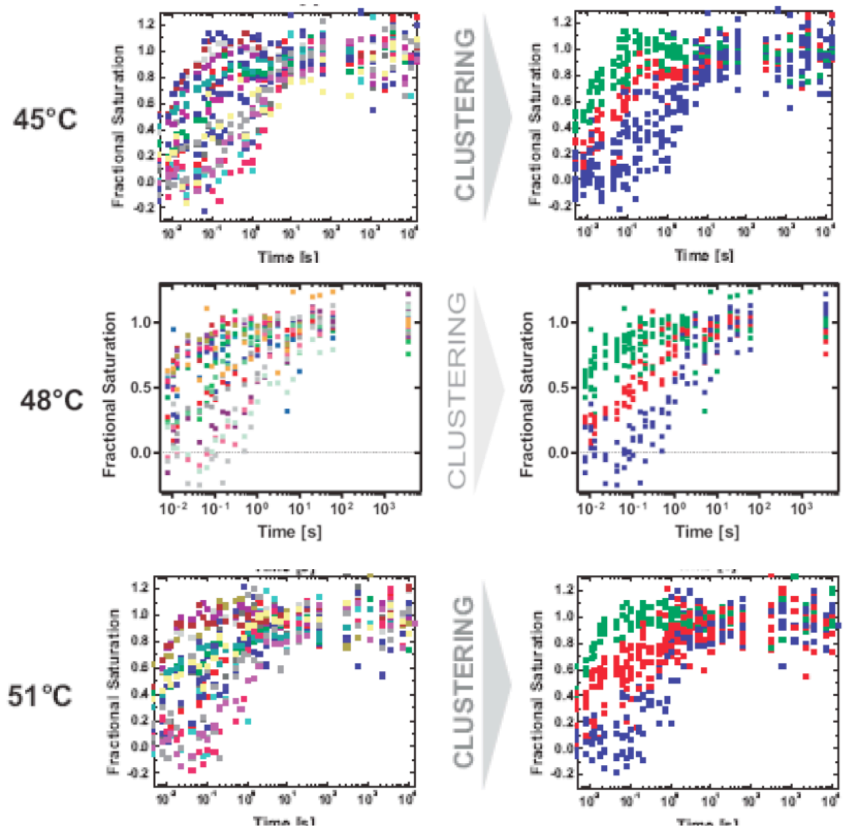

Figure S3. Clustering of time progression curves from experiments conducted between 21.5°C and 51°C. Time progression curves with individual color coding (left) are associated with three statistically significant clusters (right): fast (green), medium (red), slow (blue).
