# Supplementary material for: Mapping the Kinetic Barriers of a Large RNA Molecule's Folding Landscape
Source: PLoS One. 2014 Feb 25;9(2):e85041. doi: 10.1371/journal.pone.0085041 (PMC3934814; doi:10.1371/journal.pone.0085041)
Supplement: Figure S4 — Mean cluster centroids for folding of the L-21 Sca I RNA at the eight temperatures analyzed. The fast, medium and slow folding clusters are shown in green, red and blue, respectively. (PDF) [file pone.0085041.s004.pdf]

Supporting Information, **Figure S4**

Title: Mapping the kinetic barriers of a large RNA molecule's folding landscape

Authors: Jörg C. Schlatterer, Joshua S. Martin, Alain L. Laederach, Michael Brenowitz

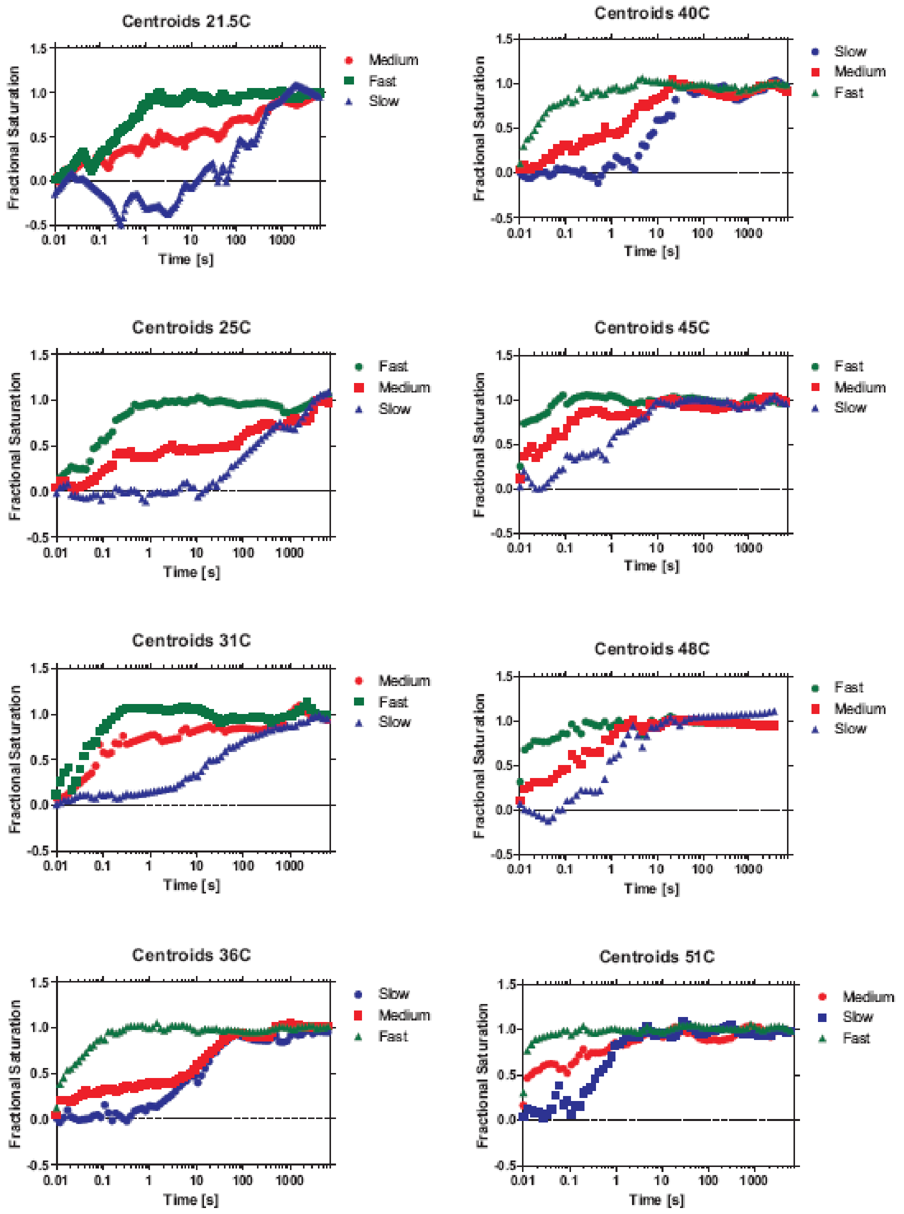

Figure S4. Mean cluster centroids for folding of the L-21 T. thermophila group I intron at eight temperatures used for this study. The fast, medium and slow folding clusters are shown in green, red and blue, respectively.
