# Supplementary material for: Mapping the Kinetic Barriers of a Large RNA Molecule's Folding Landscape
Source: PLoS One. 2014 Feb 25;9(2):e85041. doi: 10.1371/journal.pone.0085041 (PMC3934814; doi:10.1371/journal.pone.0085041)
Supplement: Figure S5 — Fitting of the medium cluster. Red dots indicate the raw data of the cluster centroid, the black line shows the bi-exponential fit of the data to equation 3. (PDF) [file pone.0085041.s005.pdf]

Supporting Information, **Figure S5**
Title: Mapping the kinetic barriers of a large RNA molecule's folding landscape
Authors: Jörg C. Schlatterer, Joshua S. Martin, Alain L. Laederach, Michael Brenowitz

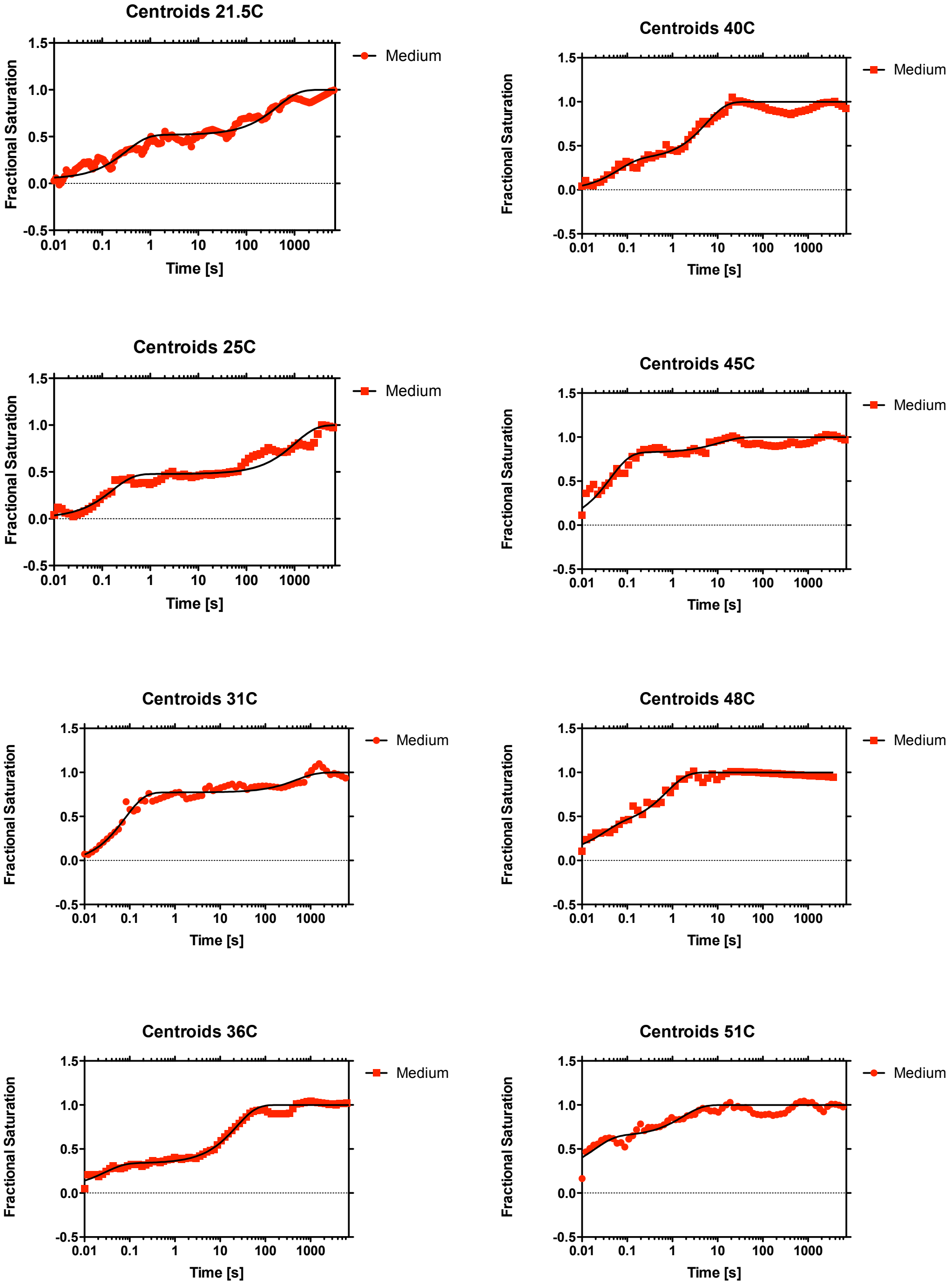

Figure S5. Fitting of the medium cluster. Red dots indicate the raw data of the cluster centroid, the black line shows the bi-exponential fit of the data to equation 3.
