# Supplementary material for: Mapping the Kinetic Barriers of a Large RNA Molecule's Folding Landscape
Source: PLoS One. 2014 Feb 25;9(2):e85041. doi: 10.1371/journal.pone.0085041 (PMC3934814; doi:10.1371/journal.pone.0085041)
Supplement: Figure S6 — Amplitudes of first (red) and second (green) phase of the intermediate cluster centroid. (PDF) [file pone.0085041.s006.pdf]

Supporting Information, **Figure S6**
Title: Mapping the kinetic barriers of a large RNA molecule's folding landscape
Authors: Jörg C. Schlatterer, Joshua S. Martin, Alain L. Laederach, Michael Brenowitz

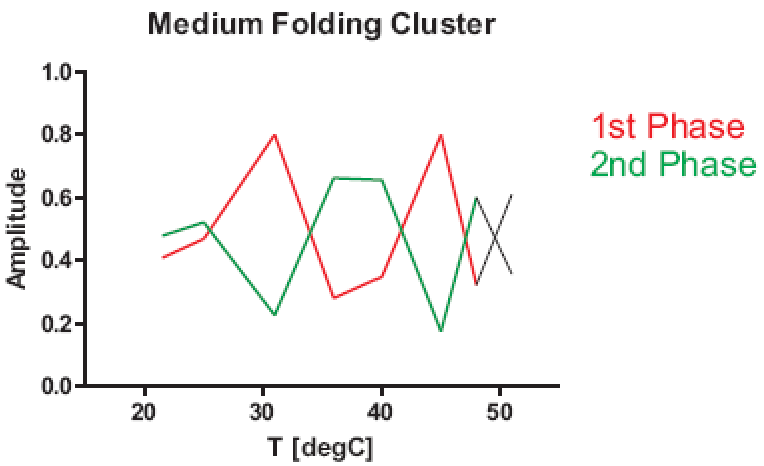

Figure S6. Amplitudes of first (red) and second (green) phase of the intermediate cluster centroid.
