# Supplementary material for: Mapping the Kinetic Barriers of a Large RNA Molecule's Folding Landscape
Source: PLoS One. 2014 Feb 25;9(2):e85041. doi: 10.1371/journal.pone.0085041 (PMC3934814; doi:10.1371/journal.pone.0085041)
Supplement: Table S1 — Rates of progression of the fast, slow and medium cluster centroids at different temperatures. (PDF) [file pone.0085041.s007.pdf]

Supporting Information, **Table S1**
Title: Mapping the kinetic barriers of a large RNA molecule's folding landscape
Authors: Jörg C. Schlatterer, Joshua S. Martin, Alain L. Laederach, Michael Brenowitz

		k-values in s^{-1}			
		**Fast**	**Slow**	**Medium (Phase 1)**	**Medium (Phase 2)**
**T in °C**	21.5	2.817	0.00308	3.101	0.002
	25	6.699	0.0031	6.044	0.001
	31	18.35	0.015	12.8	0.002
	36	27.87	0.0575	35.5	0.043
	40	20.75	0.0697	38.22	0.4
	45	76.26	0.46	50	3.081
	48	56	0.6792	36.37	1.327
	51	90.24	1.651	53.1	0.5827

		Error of k-values			
		**Fast**	**Slow**	**Medium (Phase 1)**	**Medium (Phase 2)**
**T in °C**	21.5	0.1126	0.0003	0.3197	0.0001
	25	0.4578	0.0003	1.026	0.0001
	31	1.621	0.0014	1.223	0.0004
	36	1.993	0.0041	10.55	0.0025
	40	2.239	0.0045	25.39	0.073
	45	11.49	0.0434	22.23	1.002
	48	4.68	0.0502	15.07	0.496
	51	5.56	0.0913	25.59	0.124

Table S1. Rates of progression of the fast, slow and medium cluster centroids at different temperatures.
