# Supplementary material for: Mapping the Kinetic Barriers of a Large RNA Molecule's Folding Landscape
Source: PLoS One. 2014 Feb 25;9(2):e85041. doi: 10.1371/journal.pone.0085041 (PMC3934814; doi:10.1371/journal.pone.0085041)
Supplement: Table S2 — Rates of conversion between folding species U, I1, I2, F at different temperatures. (PDF) [file pone.0085041.s008.pdf]

Supporting Information, **Table S2**
Title: Mapping the kinetic barriers of a large RNA molecule's folding landscape
Authors: Jörg C. Schlatterer, Joshua S. Martin, Alain L. Laederach, Michael Brenowitz

T in °C	k-values in s^{-1}			
	U→I1	U→I2	I1→F	I2→F
21.5	2.56E+000	1.50E+000	4.83E-003	1.66E-003
25	3.99E+000	4.45E+000	8.43E-003	1.58E-003
31	1.16E+001	5.55E+000	4.10E-002	4.20E-003
36	1.06E+001	2.06E+001	2.61E-001	4.29E-002
40	1.06E+001	1.67E+001	2.96E-002	1.29E-001
45	3.28E+001	3.43E+001	2.75E-001	4.25E+000
48	2.79E+001	3.20E+001	3.29E-001	1.35E+000
51	5.10E+001	3.56E+001	2.59E+000	1.04E+000

T in °C	Error of k-values			
	U→I1	U→I2	I1→F	I2→F
21.5	3.78E-016	2.20E-016	7.38E-019	4.46E-019
25	7.56E-016	1.07E-015	1.35E-018	3.69E-019
31	2.64E-015	6.93E-016	7.87E-018	1.11E-018
36	3.78E-016	0.00E+000	7.48E-017	2.95E-018
40	2.90E-015	3.78E-015	8.85E-018	2.95E-017
45	1.11E-014	1.21E-014	5.90E-017	5.67E-016
48	6.04E-015	8.06E-015	3.54E-017	7.87E-017
51	1.05E-015	4.19E-015	1.96E-016	1.31E-016

Table S2. Rates of conversion between folding species U, I1, I2, F at different temperatures.
