# Supplementary material for: Mapping the Kinetic Barriers of a Large RNA Molecule's Folding Landscape
Source: PLoS One. 2014 Feb 25;9(2):e85041. doi: 10.1371/journal.pone.0085041 (PMC3934814; doi:10.1371/journal.pone.0085041)
Supplement: Table S3 — Errors for the standard fitting models according to Martin et al. Briefly, errors are calculated by summing how far each data set goes below zero and normalizing according to how many points are below zero. Model 2 features the smallest error and reflects the most likely model configuration that includes U, F, and two folding intermediates (I1 and I2). (PDF) [file pone.0085041.s009.pdf]

Supporting Information, **Table S3**
Title: Mapping the kinetic barriers of a large RNA molecule's folding landscape
Authors: Jörg C. Schlatterer, Joshua S. Martin, Alain L. Laederach, Michael Brenowitz

Temperature in °C	Model 1	Model 2	Model 3
21.5	0.2618	0.1382	0.2962
25	0.1624	0.0446	0.2794
31	0.3002	0.0491	0.1018
36	0.1169	0.0198	0.1931
40	0.1512	0.0355	0.2247
45	0.1432	0.0336	0.0689
48	0.1235	0.0446	0.1265
51	0.1284	0.033	0.0642

Table S3. Errors for the standard fitting models according to Martin et al..[2] Briefly, errors are calculated by summing how far each data set goes below zero and normalizing according to how many points are below zero. Model 2 features the smallest error and reflects the most likely model configuration that includes U, F, and two folding intermediates (I1 and I2).

(2) Martin, J. S.; Simmons, K.; Laederach, A. *Algorithms* 2009, *2*, 200.
